# Hydraulic constraints to stomatal conductance in flooded trees

**DOI:** 10.1007/s00442-025-05789-y

**Published:** 2025-09-10

**Authors:** Marisa J. Brennan, Kristopher S. Criscione, Jacob A. Olichney, Junyan Ding, Yilin Fang, Nate McDowell, Brett T. Wolfe

**Affiliations:** 1 School of Renewable Natural Resources, Louisiana State University Agricultural Center, Baton Rouge, LA 70803 USA; 2 Hampton Roads Agricultural Research and Extension Center, 1444 Diamond Springs Rd, Virginia TechVirginia Beach, VA 23453 USA; 3 Atmospheric Sciences and Global Change Division, Pacific Northwest National Lab, PO Box 999, Richland, WA 99352 USA; 4 School of Biological Sciences, Washington State University, PO Box 644236, Pullman, WA 99164-4236 USA

**Keywords:** Plant stress, Process-based modeling, Root hydraulic conductance, Temperate forest, Soil–plant-atmosphere continuum, Waterlogging

## Abstract

Stomatal closure is a pervasive response among trees exposed to flooded soil. We tested whether this response is caused by reduced hydraulic conductance in the soil-to-leaf hydraulic continuum (*k*_total_), and particularly by reduced root hydraulic conductance (*k*_root_), which has been widely hypothesized. We tracked stomatal conductance at the leaf level (*g*_s_) and canopy scale (*G*_s_) along with physiological conditions in two temperate tree species, *Magnolia grandiflora* and *Quercus virginiana*, that were subjected to flood and control conditions in a greenhouse experiment. Flooding reduced *g*_s_*, G*_s_*, k*_root_ and *k*_total_. Path analysis showed strong support for direct effects of *k*_total_ on *g*_s_ and for flood duration on *k*_total_, but not *k*_root_ on *k*_total_*.* A process-based model that accounted for the *k*_total_ reduction predicted the timeseries of *G*_s_ in flood and control treatment trees reasonably well (predicted versus observed *G*_s_ *R*^2^ = 0.80 and 0.51 for *M. grandiflora* and *Q. virginiana,* respectively). However, accounting only for *k*_root_ reduction in flooded trees was insufficient for predicting observed *G*_s_ reduction. Together, these results suggest that hydraulic constraints were not limited to roots and highlight the need to account for flooding effects on *k*_total_ when projecting forest ecosystem function using process-based models.

**Supplementary Information:**

The online version contains supplementary material available at 10.1007/s00442-025-05789-y.

## Introduction

Terrestrial water is distributed heterogeneously both spatially and temporally at scales from microhabitats to biomes. Plants are generally adapted to a narrow range of water conditions outside of which their performance is impaired (Silvertown et al. 1999; Araya et al. 2011). When terrestrial plants incur drought or flood conditions, they commonly reduce their stomatal conductance (*g*_s_), which reduces both transpiration and photosynthesis (Rogers et al. 2017; McDowell et al. 2022). During droughts, reduced transpiration slows the decline of water potential within plant tissues, including leaves (Ψ_leaf_), and soil (Ψ_soil_) in the rooting zone. Water potential decline causes the total hydraulic conductance in the soil-to-leaf continuum (*k*_total_) to decline as air spaces form, blocking water flow (Venturas et al. 2017). Drought mortality is associated with reduced *k*_total_, particularly reduced stem hydraulic conductance (*k*_stem_) (Adams et al. 2017). As such, in dry conditions, plants must control *g*_s_ to balance the loss of *k*_total_ and the uptake of CO_2_ for photosynthesis. These relationships have been incorporated into process-based models of the soil–plant-atmosphere (SPA) hydraulic continuum that predict *g*_s_ with plant traits and environmental conditions (Mencuccini et al. 2019). However, SPA models have rarely been used to predict *g*_s_ under flood conditions, in part because the processes that drive *g*_s_ are less well understood than under water deficit (Liu et al. 2022).

Soil waterlogging (i.e., surface flooding) reduces oxygen availability in the rooting zone, which limits root mitochondrial respiration (Zhou et al. 2020). The effects on plant physiological function vary among species depending on traits that enable increased oxygenation of the rooting zone, such as the development of aerenchyma, adventitious roots, and hypertrophied lenticles (Fukao et al. 2019). Waterlogged plants commonly experience reduced *k*_total_, particularly root hydraulic conductance (*k*_root_) (Aroca et al. 2012). The radial pathway of *k*_root_ (i.e., between the root surface and xylem) is reduced by the regulation of aquaporins and the formation of suberin layers; responses that promote oxygen retention within the root (Aroca et al. 2012; Domec et al. 2021). Longer term waterlogging can also cause *k*_root_ decline through root dieback and reduced root growth (Pezeshki et al. 1996).

In principle, the benefit that plants derive from controlling *g*_s_ to balance CO_2_ uptake and *k*_total_ protection should apply to waterlogged scenarios as well as water deficit scenarios. If so, the reduction in *k*_root_ associated with waterlogging can be incorporated into SPA models to predict *g*_s_ responses to flooding (Liu et al. 2022). Alternatively, *g*_s_ responses to flooding could be mediated by factors independent of hydraulic constraints, in which case SPA models would be ineffective for predicting *g*_s_ in flood scenarios. In many parts of the world, the frequency of flooding is likely to increase this century (Hirabayashi et al. 2013). Ecosystem models that project responses to climatic conditions have recently been improved by incorporating SPA hydraulic processes (McDowell et al. 2013; Christoffersen et al. 2016; Anderegg and Venturas 2020). However, these models do not generally include responses to soil waterlogging.

Incorporating greater mechanistic realism, such as stomatal responses, into ecosystem models is a major goal towards improving projections (Fisher et al. 2018). If SPA models can be enabled with waterlogging responses, then incorporating them into ecosystems models would be a promising approach for projecting the effects of flooding on ecosystem dynamics. Recently, this approach was tested with the FATES-Hydro ecosystem demography model, where soil salinity and hypoxia effects were incorporated to reduce *k*_root_ and thus *g*_s_ through a SPA submodel (Ding et al. 2023). The model predicted the effects of saltwater intrusion on transpiration in coastal forests well; however, predictions under freshwater flooded conditions remain untested (Ding et al. 2023).

We tracked *g*_s_ in seedlings of two flood-sensitive tree species that were exposed to waterlogged conditions in a greenhouse experiment. We monitored physiological conditions (i.e., Ψ_leaf_, *k*_root_, *k*_stem_, and *k*_total_) and structural conditions (i.e., leaf area, root biomass, basal area) with two main objectives. First, we tested for associations between *g*_s_ regulation and tree physiological and structural conditions and used path analysis to elucidate the processes that drive stomatal responses in waterlogged conditions. Secondly, we tested the feasibility of using a SPA model to predict stomatal responses in waterlogged trees. We incorporated our measurements of physiological conditions into a SPA model that assumes plants regulate *g*_s_ to avoid the excessive loss of *k*_total_ (Sperry et al. 2016). We compared predicted and measured stomatal regulation to test the hypothesis that stomatal responses associated with waterlogging are driven by *k*_total_ reduction, and particularly *k*_root_ reduction.

## Materials and methods

### Plant material and greenhouse environmental conditions

**Fig. 1:**
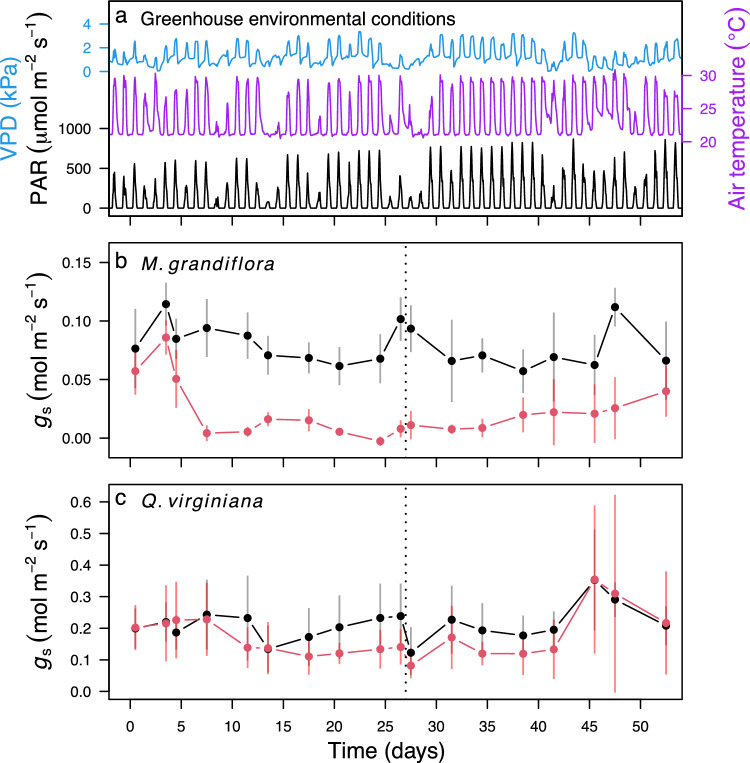
Greenhouse environmental conditions **a** and timeseries of stomatal conductance (*g*_s_) in seedlings of *Magnolia grandiflora* **b** and *Quercus virginiana* **c**. In **a** lines represent hourly averages of vapor pressure deficit (VPD; blue), air temperature (purple), and photosynthetically active radiation (PAR; black). In **b** and **c**, red and black circles represent the mean *g*_s_ among 5 plants in the flood and control treatments, respectively. Error bars extend to 1 SD and are drawn either above or below the mean to avoid overlap. The dotted line after day 26 represents the end of the flood treatment, after which flooded plants were treated the same as controls

We studied two tree species: *Magnolia grandiflora* L. (southern magnolia) and *Quercus virginiana* Mill. (live oak). Both are common within temperate deciduous forests of the southeastern United States and have been categorized as weakly tolerant to intolerant to flooding (McKnight et al. 1980). Seedlings were approximately six months old when purchased from Rennerwood, Inc. (Tennessee Colony, TX, USA) in December 2021 and transported to a greenhouse at the Louisiana State University Agricultural Center Plant Material Center in Baton Rouge, LA. Upon arrival, the seedlings were transplanted into 2.37 L containers filled with a 50–50 mixture of sand and loamy sand topsoil sourced from Clinton Township, LA, USA. At the initiation of the experiment in January 2022, seedlings of *M. grandiflora* and *Q. virginiana* were 34 ± 5 cm and 32 ± 10 cm (mean ± SD) in height and 5.9 ± 0.7 mm and 3.7 ± 0.8 mm in basal diameter, respectively. During the experiment, midday (10:00–14:00 h) greenhouse air temperature was 27.4 ± 2.8 °C, vapor pressure deficit (VPD) was 1.95 ± 0.85 kPa, and midday photosynthetically active radiation (PAR) was 423 ± 209 μmol m^−2^ s^−1^ (Fig. 1a).

### Experimental design

The experiment consisted of two measurement components: (1) a timeseries of midday *g*_s_ measurements on trees that were repeatedly measured while being subjected to experimental treatments, and (2) measurements of physiological and structural conditions on trees that were subjected to experimental treatments for varying durations and then destructively harvested. Plants were randomly assigned to be either repeatedly measured or to be destructively harvested. The timeseries of midday *g*_s_ measurements consisted of ten plants of each species that were measured for midday *g*_s_ over 51 days. At intervals of 1–5 days (Fig. 1), each plant was measured for *g*_s_ at midday (10:00–14:00 h) on three randomly selected leaves with a porometer/fluorometer (LI600, LiCor Inc., Lincoln, NE, USA). During the initial 26 days, five randomly selected plants of each species were subjected to flood conditions by placing their pots in tanks filled with tap water to 2 cm above the soil surface. Two tanks were used for the repeatedly measured plants, each with 2–3 plants per species. Dissolved oxygen was measured in one of the tanks hourly with a dissolved oxygen meter and datalogger (SDL150, Extech Nashua, NH). Dissolved oxygen content during the experiment was 4.7 ± 0.9 mg L^−1^ (mean ± SD), which is mildly hypoxic (Sasidharan et al. 2017). The remaining five plants of each species were controls that were watered to field capacity every 1–3 days throughout the experiment. After 26 days, the flooded plants were removed from the tanks and watered along with the control plants to assess post-flood recovery of *g*_s_.

Destructively harvested plants were subjected to the same flood and control treatments described above for various durations to measure physiological and structural conditions. Durations were 1, 3, 7, 10, 13, 16, 21, and 25 days. For each duration, we measured four flooded plants and two control plants of each species (*N* = 96 destructively harvested plants total). This unbalanced design was chosen to improve our ability to detect changes in conditions in the flood treatment, whereas we expected the conditions to remain unchanged with duration in the control treatment. Start and end dates of the treatments were staggered to enable replication of treatment durations, such that plants were harvested between 11-Jan-2022 and 28-Feb-2022. Eight tanks were used for the harvested plants in the flood treatment. Each tank held up to six plants at a time. Species were mixed within tanks. Flood duration was randomly assigned within and among tanks (Fig. S1). In summary, the measurements on harvested plants approximated a completely randomized design, which was used for statistical analyses.

### Physiological and structural measurements

Harvested plants were measured for the following physiological conditions: *g*_s_, light-adapted quantum efficiency (ΦPSII; i.e., the estimated proportion of absorbed light used in Photosystem II photochemistry, Krall and Edwards 1992), Ψ_leaf_, *k*_root,_ *k*_stem_ *k*_total_, and presence of hypertrophied lenticels. On three leaves per plant, we used a porometer/fluorometer (LI600, LiCor Inc.) to measure *g*_s_ and Φ_PSII_. Following the porometer/fluorometer measurements, Ψ_leaf_ was measured on three leaves with a pressure chamber (1505D-EXP; PMS Instrument Co., Albany, OR, USA). All leaves were removed from each plant with razors at the petiole base and total leaf area was measured with an area meter (LI3100, LiCor Inc.). Basal diameter was measured with calipers and used to calculate basal area assuming that stem cross sections were circular. We visually inspected the base of each harvested plant for the presence or absence of hypertrophied lenticles.

We used the vacuum chamber technique described by Kolb et al. (1996) to measure.

*k*_root_ and *k*_stem_ (Fig. S2). This technique is well-suited for measuring responses to stress in both *k*_root_ and *k*_stem_ (Kursar et al. 2009; Torres-Ruiz et al. 2015; Venturas et al. 2018). Stems were excised approximately 5 cm above the root collar with pruning shears while the plants were submerged in tap water to avoid xylem air entry. For *k*_root_, the pot, including soil and intact root system, was placed in a vacuum chamber with the stump protruding out of the chamber through a hole that was sealed with rubber gaskets and parafilm to achieve vacuum pressure within the chamber. The cut end of the stump was recut with a fresh razor and connected via tubing to a reservoir and graduated pipette filled with 10 mM KCl solution that was filtered to 0.2 μm and degassed. Vacuum pressure was applied in a sequence of 0, 20, 40, and 0 kPa for at least five minutes at each pressure. The volume of solution in the pipette was tracked to obtain flow rate at each pressure. Hydraulic conductance was calculated with linear regression as the slope of the relationship between flow rate and pressure. Hydraulic conductance was standardized by basal area to obtain *k*_root_*.* Although the soil could conflate our assessment of *k*_root_, this effect was likely negligible because hydraulic conductivity is orders of magnitude higher in saturated soil than in roots (Sperry et al. 1998). To measure *k*_stem_, we used the same methods as for *k*_root_ by placing the stem (with leaves removed) in the vacuum chamber with the cut end protruding. Note that *k*_stem_ and *k*_root_ are not scaled by pathlength (to obtain conductivity) because the pressure gradient was applied to the entire surface of stem and root.

For each harvested plant, we calculated *k*_total_ by combining leaf-level measurements of transpiration (E) and Ψ_leaf_, where *k*_total_ = E / (Ψ_leaf_ – Ψ_soil_) (e.g., Limousin et al. 2013). Because soils were maintained near saturation and at saturation in the control and flood treatments, respectively, we used Ψ_soil_ = 0 for all samples. E was output from the LI600 porometer, which was measured on three leaves per plant and averaged. The E value assumes negligible leaf boundary layer resistance, which was valid in our experiment because ceiling fans in the greenhouse continually moved air and the seedlings had minimal canopy development that would impede air movement. To scale *k*_total_ by basal area for comparison with *k*_stem_ and *k*_root_ we multiplied E (which is scaled by leaf area) by total leaf area and divided by stem basal area.

After measuring *k*_root_, we carefully rinsed the soil from the roots, using a 2-mm mesh screen to collect disconnected roots. Dead roots were distinguished by their color and friability (Powers and Peréz-Aviles 2013) and excluded from analyses. Fine roots (< 2 mm diameter) and coarse roots were separated and dried at 65 °C to obtain dry mass. Root dry mass was scaled by basal area to obtain fine root dry mass per basal area (DM_fine_root_) and coarse root dry mass per basal area (DM_coarse_root_). Similarly, total leaf area was divided by basal area to obtain leaf area per basal area (LA:BA).

### Statistical analyses

All statistical analyses were completed in R 4.3.0 (R Core Team 2023). To test for an effect of flooding (days 0–26 in Fig. 1) on the repeatedly measured plants, we used linear mixed effect models in the package lme4 (Bates et al. 2015). Treatment, duration, and their interaction were fixed effects. Individual plant was a random effect. We used a likelihood- ratio test between the full model and a model with the treatment effects removed to test for the significance of a flood effect on g_s_. Separately, we used the same model structure to test for an effect of treatment during the post-flood period (days 27–52 in Fig. 1).

In the harvested plants, we tested whether the rate of change in physiological conditions (i.e., g_s_, Φ_PSII_, and Ψ_leaf_, *k*_total_, *k*_stem_, *k*_root_) and structural conditions (i.e., DM_fine_root_, DM_coarse_root_, LA:BA, and basal area) differed between flood and control treatments with linear models of the interaction between treatment and duration. For *g*_s_, Φ_PSII_, and Ψ_leaf_, which had three measurements per plant, values were averaged within plant prior to analysis. For these tests, we used a shared intercept term because at the model intercept (i.e., duration of zero) the flood treatment had not yet been applied, so physiological conditions were assumed to be equal. To account for the unbalanced design, we used Type II ANOVA tests in the car package (Fox and Weisberg 2019) to test for the significance of the treatment by duration interaction. We used logistic regression to analyze the relationship between flood duration and the presence/absence of hypertrophied lenticels. None of the control-treatment plants formed hypertrophied lenticels, so they were excluded from this analysis. Pairwise Spearman correlations were used to evaluate the bivariate relationships between physiological and structural conditions within plants. For each species, we tested each correlation with plants from both treatments (i.e., control and flood) combined and in isolation.

We used path analysis to assess the causal relationships between *g*_s_, physiological conditions, and environmental conditions. A set of four candidate models was tested with data from the harvested plants of each species. Each model included direct effects of conditions known to affect *g*_s_ (i.e., PAR, VPD, and *k*_total_) and the cascading effects of flood duration on *k*_root_ and *k*_root_ on *k*_total_. The models varied in their inclusion of paths from flood duration to *k*_total_, representing a hydraulic constraint separate from *k*_root_, and path from flood duration to *g*_s_, representing a constraint on *g*_s_ separate from hydraulics. Model selection followed the protocol suggested by Garrido et al. (2022). The models were ranked by Akaike weights calculated from the Akaike information criterion index corrected for the sample size (AICc). The AICc balances goodness of fit with model complexity and is adjusted for finite samples to ensure accurate comparisons across different datasets or study sizes. The Akaike weights represent the relative probability that each model is the best one given the data. In the final model, the path coefficients (*β*; i.e., an indicator of relationship strength and direction between two variables) were calculated with weighted averages from the highest-ranking models that accounted for ≥ 55% Akaike weight. For path analysis, variables were transformed to improve multivariate normality, including *g*_s_, *k*_total_, and flood duration. All variables were zero centered and scaled by SD. Path coefficients were estimated with maximum likelihood in the R package lavaan (Rosseel 2012).

### Soil–plant-atmosphere hydraulic modeling

We used the hydraulic model developed by Sperry et al. (2016) to test whether the stomatal responses that we observed in flooded plants could be predicted by observed reductions in *k*_root_ and *k*_total_. The model has accurately predicted stomatal behavior in a wide variety of trees and environmental conditions, but to our knowledge it has not been applied to flood scenarios (Sperry et al. 2016; Wolfe et al. 2016). The model assumes that plants control *g*_s_ to balance the supply of water available for transpiration with the atmospheric demand for water (i.e., VPD). Water supply is a function of Ψ_soil_ and hydraulic conductance in the rhizosphere, roots, stems, and leaves. Hydraulic conductance in each of these components is defined by a vulnerability curve that responds to water potential and by the component’s proportional contribution to the maximum total hydraulic conductance (*k*_total,max_, i.e., *k*_total_ when Ψ_soil_ is high and VPD is low). The model predicts steady-state canopy vapor conductance (*G*) given a timeseries of VPD and Ψ_soil_. *G* is standardized by tree basal area and includes boundary layer resistance. For our purposes, since boundary layer resistance was negligible, we assumed that *G* was equal to canopy-scale stomatal conductance (*G*_s_). We converted measurements of *g*_s_ to *G*_s_ by multiplying *g*_s_ by total leaf area and dividing by basal area.

Details of our modeling approach are in described in Methods S1. Briefly, our approach had three steps. First, we fit the model to the timeseries of repeatedly measured control-treatment plants (i.e., the plants represented by black circles in Fig. 1). This included inputting environmental conditions, including soil texture, Ψ_soil_, and VPD; and plant traits including vulnerability-curve coefficients, and the partitioning of *k*_total,max_ among roots, stems, and leaves (Table S1). We adjusted two unknown parameters, *k*_total,max_ and maximum *G*, to find the best fit between predicted and measured *G*_s_, following the approach of Sperry et al. (2016). We performed a sensitivity analysis by varying inputted vulnerability-curve coefficients to test how assumptions of vulnerability segmentation affected model outputs.

Next, we predicted *G*_s_ in the timeseries of repeatedly measured flood-treatment plants (i.e., the plants represented by red circles in days 0–26 in Fig. 1) by incorporating the effects of flooding on hydraulic traits that were observed in the harvested plants (Table 1). We compared two parameterizations; both used the same parameter values as in the control treatment plants and had an additional parameter that adjusted *k*_total,max_ as a function of flood duration. One parametrization reduced *k*_total,max_ by the amount predicted by measurements of *k*_root_ and one reduced *k*_total,max_ by the amount predicted by measurements of *k*_total_. Lastly, we tested the model’s performance in predicting *G*_s_ across flood and control treatments by fitting least squares linear regressions through observed versus model-predicted values. We used *R*^2^ as an indicator of goodness of fit and the slope and intercept as indicators of bias.

**Table 1:** Results from linear models for the effects treatment (flood versus control) on the change in physiological conditions over time

Condition	Intercept	Control treatment slope	Flood treatment slope	*P*	*R*^2^
*M. grandiflora*					
* g*_s_ (mmol m^−2^ s^−1^)	0.065 ± 0.010	0.0019 ± 0.0009	− 0.0030 ± 0.0007	**1.8e− 6**	0.44
Φ_PSII_ (unitless)	0.614 ± 0.035	0.0011 ± 0.0032	− 0.0129 ± 0.0026	**4.8e− 6**	0.42
Ψ_leaf_ (MPa)	− 0.738 ± 0.037	0.0006 ± 0.0034	− 0.0004 ± 0.0028	0.95	0.002
*k*_total_ (mol m^−2^ s^−1^ MPa)	3.96 ± 0.49	0.103 ± 0.045	− 0.178 ± 0.037	**4.1e− 8**	0.51
*k*_stem_ (mol m^−2^ s^−1^ MPa)	28.36 ± 5.45	0.457 ± 0.499	0.2667 ± 0.418	0.64	0.02
*k*_root_ (mol m^−2^ s^−1^ MPa)	5.87 ± 0.43	0.005 ± 0.039	− 0.091 ± 0.033	**9.4e− 3**	0.18
DM_fine_root_ (kg m^−2^)	100.6 ± 9.2	0.79 ± 0.83	− 1.46 ± 0.70	**0.013**	0.14
DM_coarse_root_ (kg m^−2^)	58.5 ± 6.0	0.64 ± 7.67	7.74 ± 6.47	0.11	0.05
LA:BA (m^2^ m^−2^)	1839 ± 97	− 5.7 ± 8.9	− 3.0 ± 7.5	0.81	0.00
Basal area (mm^−2^)	21.8 ± 1.2	0.16 ± 0.11	0.03 ± 0.10	0.37	0.00
***Q. virginiana***					
* g*_s_ (mmol m^−2^ s^−1^)	0.232 ± 0.034	0.0021 ± 0.0030	0.0022 ± 0.0025	0.66	0.02
Φ_PSII_ (unitless)	0.708 ± 0.017	− 0.0007 ± 0.0016	− 0.0035 ± 0.0013	**0.025**	0.11
Ψ_leaf_ (MPa)	− 1.565 ± 0.124	0.010 ± 0.0113	0.0084 ± 0.0094	0.58	0.02
*k*_total_ (mol m^−2^ s^−1^ MPa)	1.49 ± 0.52	0.221 ± 0.051	0.043 ± 0.040	**2.6e− 4**	0.31
*k*_stem_ (mol m^−2^ s^−1^ MPa)	26.31 ± 3.50	− 0.212 ± 0.315	0.114 ± 0.265	0.55	0.03
*k*_root_ (mol m^−2^ s^−1^ MPa)	3.32 ± 0.84	0.251 ± 0.084	0.143 ± 0.065	**0.012**	0.17
DM_fine_root_ (kg m^−2^)	159.9 ± 23.2	4.69 ± 2.10	1.94 ± 1.78	0.093	0.06
DM_coarse_root_ (kg m^−2^)	609.3 ± 84.7	1.05 ± 0.54	7.74 ± 6.47	0.43	0.00
LA:BA (m^2^ m^−2^)	599 ± 77	− 1.7 ± 7.0	− 5.8 ± 5.9	0.59	0.00
Basal area (mm^−2^)	10.3 ± 1.1	− 0.02 ± 0.10	− 0.11 ± 0.08	0.42	0.00

Values are estimates ± 1SE. Slopes are units per day. *P* refers to Type II test for treatment by time interaction. Those in bold are < 0.05. *R*^2^ is the coefficient of determination for the model

## Results

Among the repeatedly measured plants, *M. grandiflora g*_s_ was stable in the control treatment throughout the experiment; however, in the flood treatment *g*_s_ declined during the first six days and remained low (Fig. 1b). Between days 6 and 26, *g*_s_ was reduced on average by 91% in the flood treatment compared to the controls. Overall, the effect of flooding on *g*_s_ was highly significant (*P* < 0.001; Fig. 1b). When the flood treatment was stopped (i.e., day 27 onward in Fig. 1b), *g*_s_ remained on average 74% lower in the flooded plants compared to the controls (*P* < 0.001). Flooding had less of an effect on *Q. virginiana g*_s_. Between days 6 and 26, *g*_s_ was reduced on average by 31% in the flood treatment compared to the controls (*P* < 0.001; Fig. 1c). When the flood treatment was stopped, *g*_s_ in *Q. virginiana* mostly recovered. On average, it was only 15% lower than the controls, which was not a significant difference (*P* = 0.29; Fig. 1c).

**Fig. 2:**
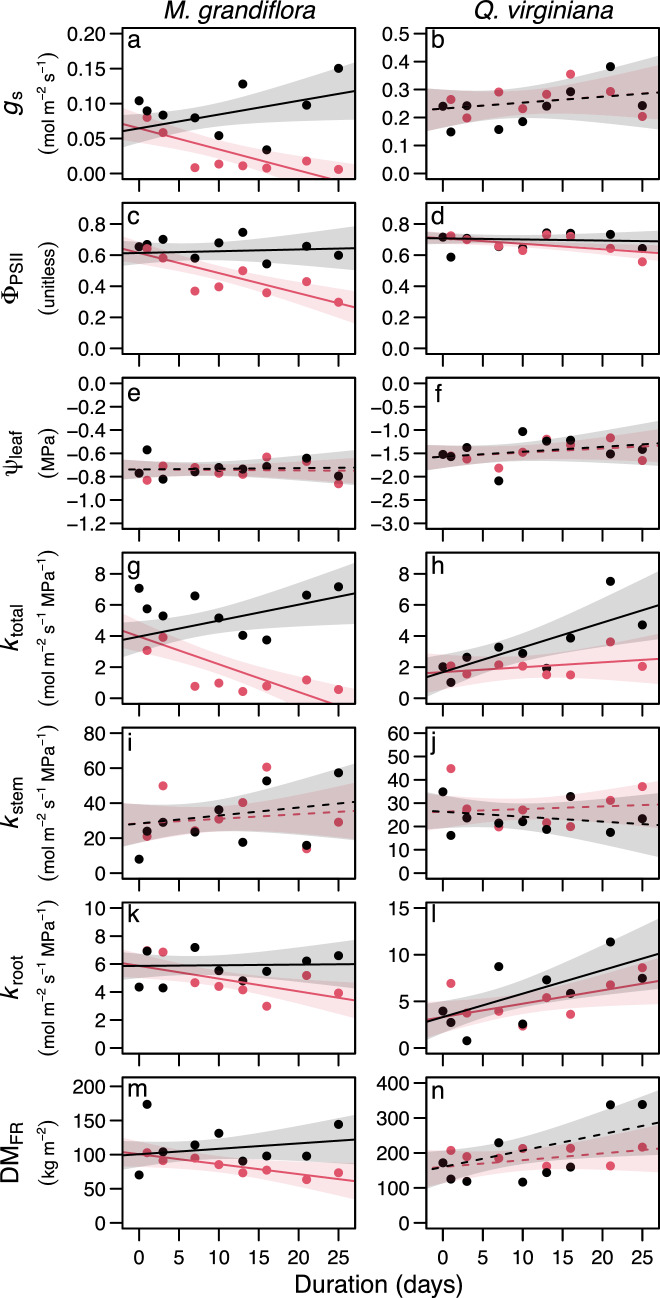
Responses of physiological conditions to flood and control treatments in *Magnolia grandiflora* (a,c,e,g,i,k,m) and *Quercus virginiana* (b,d,f,h,j,l,n). Red and black symbols represent the flood and control treatments, respectively. Each circle represents the mean of 2–4 plants. Lines represent outputs from linear models with the treatment by duration interaction as the independent variable and the physiological condition as the dependent variable. Shaded regions are the 95% confidence intervals. Solid lines indicate that the flood and control treatments had significantly different slopes (*P* < 0.05), otherwise the lines are dashed

Among the harvested plants, there were no significant differences in Ψ_leaf_ or *k*_stem_ between control and flood treatments in either species, or for *g*_s_ in *Q. virginiana* (Fig. 2, Table 1). All other physiological conditions measured (i.e., Φ_PSII_, *k*_total_, *k*_root_) were affected by the flood treatment in both species. In general, the effects of flooding were stronger in *M. grandiflora* than in *Q. virginiana* (Fig. 2; Table 1). In *Q. virginiana*, most physiological conditions did not decrease with duration in the flood treatment; rather, they had lower rates of increase than in the control treatment. In contrast, *M. grandiflora* had a reduction (i.e., negative slope with duration) in most physiological conditions in the flood treatment, but not in the controls (Fig. 2; Table 1).

Structural conditions, including LA:BA, DM_coarse_root_, and basal area did not change with duration or vary between the flood and control treatments (Table 1, Fig. S4). However, DM_fine_root_ in *M. grandiflora* decreased with duration in the flood treatment and did not change with duration in the control treatment (Fig. 2; Table 1). Flooded plants of both *M. grandiflora* and *Q. virginiana* developed hypertrophied lenticels (Fig. S5). Logistic regression estimates for the flood duration at which 50% of plants produced hypertrophied lenticels were 15.3 and 23.5 days in *M. grandiflora* and *Q. virginiana*, respectively (Fig. S5, Table S2).

**Fig. 3:**
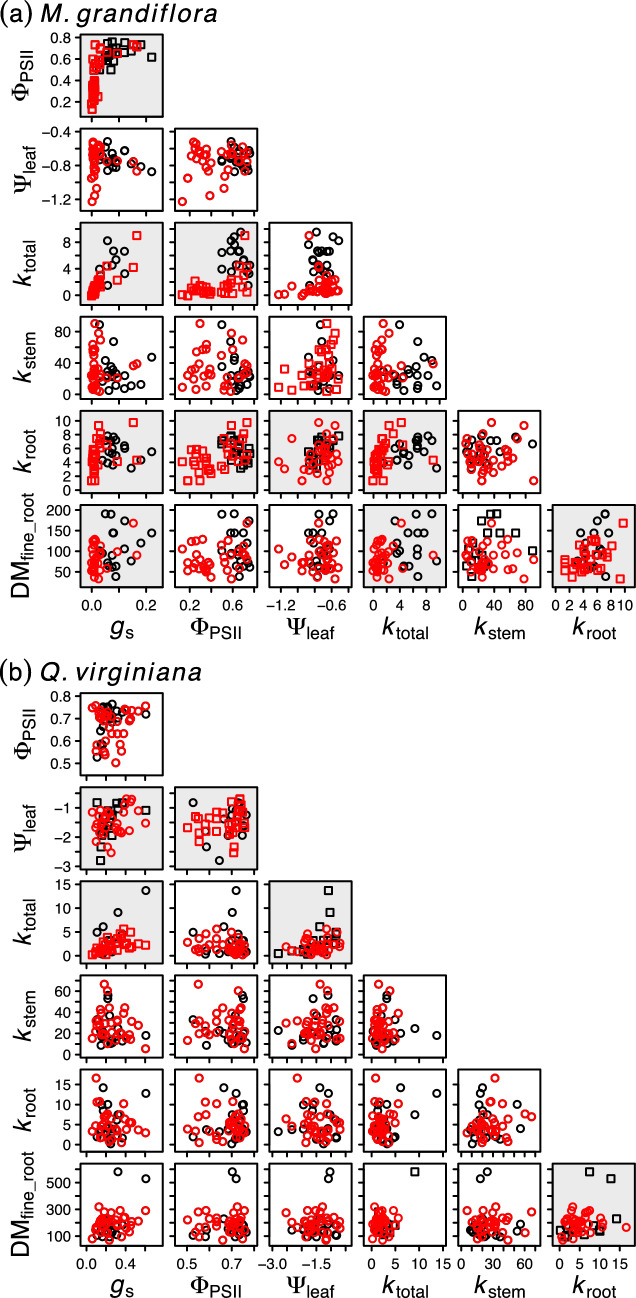
Bivariate relationships between physiological conditions in harvested plants of *Magnolia grandiflora* **a** and *Quercus virginiana* **b**. Red and black symbols represent individual plants in the flood and control treatments, respectively. Plants are represented as squares when the Spearman correlation coefficient within treatment differed from zero (*P* < 0.05) and otherwise as circles. Shaded panels indicate that the Spearman correlation coefficient for treatments combined differed from zero. Correlation coefficients are given in Table S3. Units of measurement are given in Table 1

Among the pairwise correlations between physiological and structural conditions, in the flood treatment *M. grandiflora g*_s_ was positively correlated with *k*_root_, Φ_PSII_, and *k*_total_ (Fig. 3a, Table S3). Furthermore, Φ_PSII_ was positively correlated with *k*_root_ and *k*_total_; Ψ_leaf_ was positively correlated with *k*_stem_; *k*_total_ was positively correlated with *k*_root_; and *k*_root_ root was positively associated with DM_fine_root_. In contrast, in the control treatment, *M. grandiflora g*_s_ was associated only with Φ_PSII_; however, Φ_PSII_ was negatively correlated with *k*_root_, Ψ_leaf_ was positively correlated with *k*_root_, and *k*_stem_ was positively correlated with DM_fine_root_. In flooded *Q. virginiana*, *g*_s_ was positively correlated only with *k*_total_ and Φ_PSII_ was positively correlated with Ψ_leaf_ (Fig. 3b, Table S3). In the control treatment, *Q. virginiana k*_total_ was positively correlated with Ψ_leaf_, and DM_fine_root_ was positively associated with *k*_root_.

**Fig. 4:**
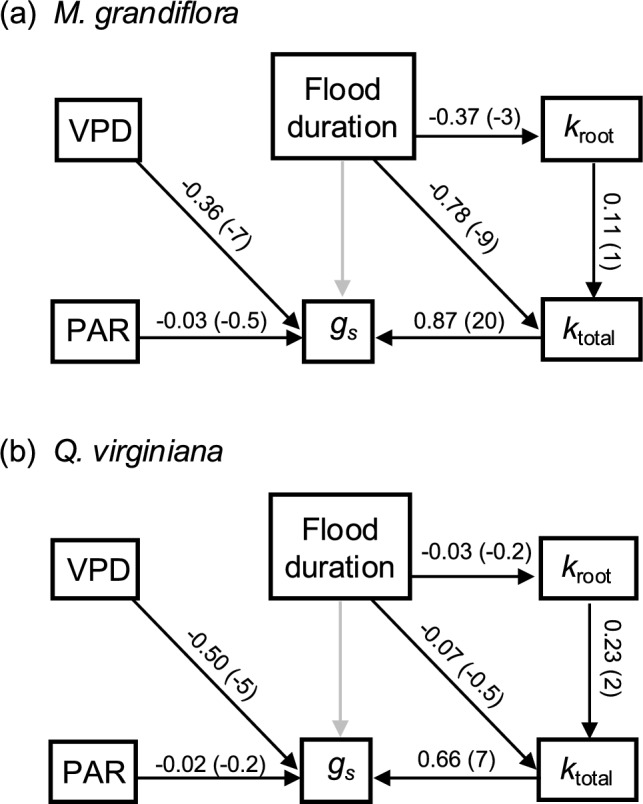
Path analysis models for the effects of environmental conditions and their physiological mediators on stomatal conductance (*g*_s_) for *Magnolia grandiflora* **a** and *Quercus virginiana* **b**. Boxes and arrows represent variables and hypothesized causality, respectively. Variables include flood duration, root hydraulic conductance (*k*_root_), total soil-to-leaf hydraulic conductance (*k*_plant_), photosynthetically active radiation (PAR), and vapor pressure deficit (VPD). Numbers indicate weighted means of path coefficients (*β*) and *β*/SE in parentheses. Gray arrows represent relationships that were not included in the final model (Table S4)

The best-fitting path model (i.e., accounting for ≥ 55% Akaike weight) for *M. grandiflora* was the model with a direct path from flood duration to *k*_total_ but not from flood duration to *g*_s_ (Table S4). The model showed strong support (i.e., *β* and *β*/SE far from zero) for direct effects of flood duration on *k*_root_ (*β* = − 0.37*, β*/SE = − 3) and *k*_total_ (*β* = − 0.78*, β*/SE = − 9), but only weak support for an effect of *k*_root_ on *k*_total_ (*β* = 0.11*, β*/SE = 1) (Fig. 4a). For *Q. virginiana*, the best fitting model included the model with a direct effect of flood duration on *k*_total_ and the model with only the direct effect of flood duration on *k*_root_ (Table S4). The model showed strong support for an effect of *k*_root_ on *k*_total_ (*β* = 0.23*, β*/SE = 2), but weak support for effects on flood duration on *k*_root_ (*β* = − 0.03*, β*/SE = − 0.2) and *k*_total_ (*β* = − 0.07*, β*/SE = − 0.5) (Fig. 4b). For both species, *k*_total_ and VPD were the main drivers of *g*_s_ variation. PAR was relatively low throughout the experiment because of the seasonally low solar zenith angle (Fig. 1), and it had no effect on *g*_s_ variation (Fig. 4).

**Fig. 5:**
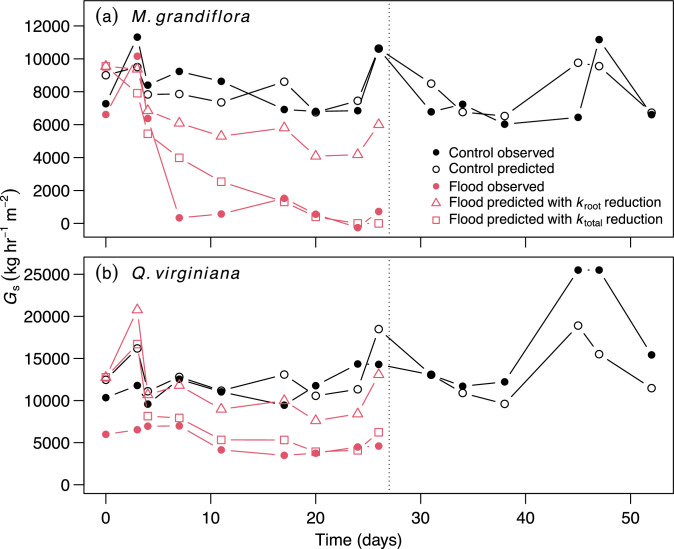
Timeseries of observed and soil–plant–atmosphere model-predicted canopy-scale stomatal conductance (*G*_s_, kg water hr^−1^ m^−2^ basal area) for *Magnolia grandiflora* **a** and *Quercus virginiana* **b**. The legend in **a** also applies to **b**. The dotted line after day 26 represents the end of the flood treatment. In the flooded plants, reductions of hydraulic conductance in roots (*k*_root_) and whole plants (*k*_total_) were simulated using the linear models obtained from harvested plants (Table 1)

**Fig. 6:**
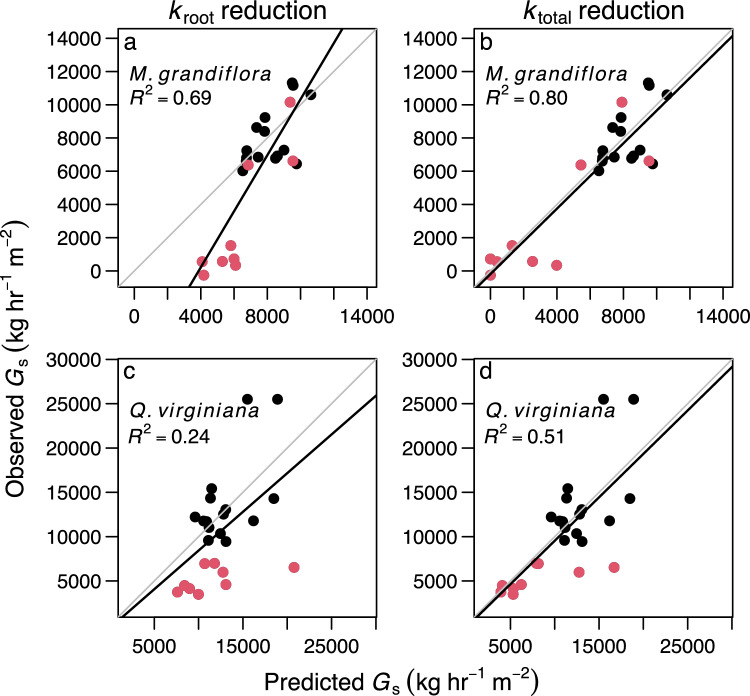
Observed canopy-scale stomatal conductance (*G*_s_, kg water hr^−1^ m^−2^ basal area) versus soil–plant–atmosphere model-predicted *G*_s_. Black and red circles represent the means of five plants repeatedly measured in the control and flood treatments, respectively. In the flooded plants, reductions of hydraulic conductance in roots (*k*_root_, panels **a** and **c**) and whole plants (*k*_total_, panels **b** and **d**) were simulated using the linear models obtained from harvested plants (Table 1). Gray lines are 1:1. Black lines and *R*^2^ values represent least squares regressions fit through all points. Regression fits are summarized in Table S5

The SPA hydraulic model predicted *G*_s_ reasonably well when fit to the control plants, with mean absolute errors between predicted and observed *G*_s_ of 14 and 21% for *M. grandiflora* and *Q. virginiana*, respectively (Figs. 5, 6). With these reasonable model fits in the control plants, we were able to predict the effects of flood-induced reductions in *k*_root_ and *k*_total_ on *G*_s_. In both *M. grandiflora* and *Q. virginiana*, measured reductions in *k*_root_ were not sufficient to predict the observed reduction in *G*_s_ (red circles are below the 1:1 line in Fig. 6a, c). In contrast, measured reductions in *k*_total_ predicted *G*_s_ in the flooded plants well (red circles are near the 1:1 line in Fig. 6b, d). The models that incorporated the effects of flooding on *k*_total_ had better fit than those that incorporated *k*_root_ in isolation (*R*^2^ = 0.80 vs. 0.69 for *M. grandiflora* and *R*^2^ = 0.51 vs. 0.28 for *Q. virginiana;* Fig. 6; Table S5). Results were insensitive to the hydraulic vulnerability assumptions, including varying vulnerability by 20% and introducing hydraulic segmentation (Table S6).

## Discussion

Compared to control conditions, flooding reduced *g*_*s*_, *G*_s_*,* Φ_PSII_, *k*_total_ and *k*_root_, but not Ψ_leaf_ or *k*_stem_ in seedlings of *M. grandiflora* and *Q. virginiana* (Figs. 1, 2). Path models indicated that the *g*_*s*_ response to flooding was likely mediated by hydraulic constraints (Fig. 4). When we parameterized a SPA hydraulic model with the *k*_total_ reduction caused by flood conditions, it predicted the timeseries of *G*_s_ in flooded seedlings reasonably well (Figs. 5, 6). However, accounting for *k*_root_ reduction alone was insufficient for predicting measured *G*_s_ reduction in flooded trees, suggesting that hydraulic constraints are not limited to roots and may also occur in leaves. Our results demonstrate the utility of SPA hydraulic models for projecting the effects of flooding on forest ecosystem function and the need to consider *k*_total_ reduction during flooding.

### Physiological responses to waterlogging

Reduced *g*_s_ is commonly observed in trees subjected to flood conditions, with the timing and extent of *g*_s_ reduction varying widely among species (Kozlowski 1984; Pezeshki and Anderson 1997; Lopez and Kursar 2003; Aroca et al. 2012). These leaf-level effects are likely mediated by hydraulic and chemical signals that originate at the site of waterlogging in the roots, but this communication pathway is poorly understood (Vartapetian and Jackson 1997; Jackson 2002). Reduced *g*_s_ in waterlogged trees is often correlated with reduced *k*_root_ (Andersen et al. 1984; Schmull and Thomas 2000; Islam and Macdonald 2004; Aroca et al. 2012; Karlova et al. 2021), supporting the hypothesis that *g*_s_ reduction is a response to hydraulic constraints. Consistent with these results, we found concurrent reductions in *g*_s_ and *k*_root_ among *M. grandiflora* seedlings after 6 days of flooding (Figs. 2, 3; Table 1). In *M. grandiflora*, *k*_root_ reduction may have, at least partially, been caused by fine root dieback, as evidenced by the reduction in DM_fine_root_ and the correlation between DM_fine_root_ and *k*_root_ (Figs. 2, 3; Table 1). However, this result does not exclude the possibility that *k*_root_ was affected by aquaporin activity (Domec et al. 2021) or embolisms (Li et al. 2015).

In both species, *k*_total_ increased with duration in the control treatment, while in the flood treatment it decreased (*M. grandiflora*) or remained the same (*Q. virginiana*) (Fig. 2, Table 1). Since *k*_root_ followed the same pattern (Fig. 2, Table 1) and since *k*_total_ and *k*_root_ were positively correlated (Fig. 3, Table S3), it is possible that *k*_root_ limited *k*_total_ in flooded plants compared to control plants. Unless *k*_root_ reduction was compensated by higher *k*_stem_ or leaf hydraulic conductance (*k*_leaf_), it would cause *k*_total_ reduction. However, for both species, the divergence in *k*_root_ between flood and control treatments was less than that of *k*_total_ (Fig. 2, Table 1). Since *k*_stem_ was unaffected by flooding in either species, the remaining impact on *k*_total_ likely occurred in the leaves. We did not measure *k*_leaf_, however *k*_leaf_ is known to be highly dynamic in response to environmental conditions such as PAR, VPD, and Ψ_soil_ (Prado and Maurel 2013). In loblolly pine (*Pinus taeda*), 35 days of flooding reduced aquaporin activity in leaves, contributing to a 47% reduction in *k*_total_ (Domec et al. 2021). Together, these results suggest that *k*_leaf_ reduction may be a common response to flooding. Furthermore, our path analysis showed that *g*_s_ was highly responsive to *k*_total_, but *k*_root_ did not mediate *k*_total_ (Fig. 4). Instead, when flooding affected *k*_total_ it was through a direct link (Fig. 4a), which is likely explained by a reduction in *k*_leaf_.

Midday Ψ_leaf_ was unaffected by flooding in either species (Fig. 2, Table 1), indicating that stomatal control compensated for *k*_total_ reduction, resulting in Ψ_leaf_ homeostasis. Flooding also had no effect on Ψ_leaf_ in similar experiments on tomato plants (*Lycopersicon esculentum*) (Bradford and Hsiao 1982; Else et al. 2009), pea plants (*Pisum sativum*) (Zhang and Davies 1986), sweetgum seedlings (*Liquidambar styraciflua*) (Pezeshki and Chambers 1985), and oak seedlings (*Quercus robur* and *Quercus petraea*) (Schmull and Thomas 2000). In contrast, flooding reduced midday Ψ_leaf_ compared to well-watered controls in beech seedlings (*Fagus sylvatica*) by 16% across three months (Schmull and Thomas 2000), in apricot saplings (*Prunus armeniaca*) by 67% after 50 h (Nicolás et al. 2005), and in almond seedlings (*Amygdalus communis*) by 125% after 7 days (Sanchez-Blanco et al. 1994). Flooding also increased midday Ψ_leaf_ in elm seedlings, by 18% across 60 days in *Ulmus laevis* and by 12% across 45 days in *U. minor* (Li et al. 2015). Ψ_leaf_ regulation in response to soil drying varies among plant species across a spectrum of isohydry to anisohydry (Klein 2014; Meinzer et al. 2016). Although Ψ_leaf_ regulation under waterlogging has been studied far less than under soil drying, our results combined with those in the literature suggest that a similar spectrum of Ψ_leaf_ regulation exists in response to soil waterlogging. Whether or not species’ positions on a spectrum of Ψ_leaf_ regulation in response to drought and waterlogging are associated is unclear. Both of our focal species can be described as partially isohydric in response to drought (Cooper et al. 2018; Vastag et al. 2020). Considering that they also maintained Ψ_leaf_ in response to waterlogging (Fig. 2), this limited sample suggests an association between degrees of drought and waterlogging isohydricity.

In *M. grandiflora*, flooding reduced Φ_PSII_ by 54% compared to controls, while in *Q. virginiana,* flooding reduced Φ_PSII_ by only 9% (Fig. 2, Table 1). For *M. grandiflora*, Φ_PSII_ and *g*_s_ reduction occurred concurrently (Fig. 2a,c) and the two physiological conditions were highly correlated among plants (Fig. 3, Table S3). It is possible that stomatal closure was a response to Φ_PSII_ reduction, since stomata close in response to reduced carbon assimilation (Wang et al. 2020). However, it is more likely that the reduction in Φ_PSII_ was a result of stomatal closure. Reduced *g*_s_ would lead to reduced internal CO_2_ concentration (*c*_i_). Reducing *c*_i_ either by flooding or by experimentally reducing ambient CO_2_ concentration has been shown to affect Φ_PSII_ similarly, likely because NADP^+^ (nicotinamide adenine dinucleotide phosphate) regeneration slows when *c*_i_ is low (Else et al. 2009). Although we did not measure other photosynthetic parameters, flooding has also been shown to affect maximum quantum yield and ribulose bisphosphate activity in *L. esculentum* (Else et al. 2009) and *P. taeda* (Domec et al. 2021).

### Predicting the effects of waterlogging on stomatal control with SPA hydraulic models

SPA hydraulic models are useful for predicting *G*_s_ or *g*_*s*_ with sets of plant traits and environmental conditions. They parametrize *k*_total_ with vulnerability curves that reduce rhizosphere conductance, *k*_root_, *k*_stem_, and *k*_leaf_ as a function of their respective water potentials under scenarios of Ψ_soil_ and VPD (Mencuccini et al. 2019). Because they generally do not account for other factors that influence *k*_total_, such as soil waterlogging, their utility is limited to water deficit scenarios (Liu et al. 2022). To predict responses to flooding, we incorporated into an existing SPA hydraulic model a parameter that reduced *k*_total_ as a function of time in waterlogged soil. This arrangement was able to predict the contrasting patterns in *G*_s_ that we observed in timeseries of flooded seedlings of *M. grandiflora* and *Q. virginiana* (Figs. 4bd, 5bd). A recent SPA hydraulic model developed for predicting the effects of soil waterlogging on stomatal behavior incorporated only the effect of waterlogging on *k*_root_ to parameterize a *k*_total_ response (Liu et al. 2022). Similarly, the recent inclusion of waterlogging into the SPA hydraulic submodel of the FATES-Hydro ecosystem demography model incorporated only effects on *k*_root_ (Ding et al. 2023). These approaches follow the common assumption that the effects of waterlogging on plant hydraulics are limited to roots. However, our SPA model runs that accounted for the reduction in *k*_root_ alone did not generate the *G*_s_ response that we observed in flooded plants (Figs. 5, 6). These results suggest that SPA model development aimed at projecting *G*_s_ in flooded plants should account for the effects of waterlogging on hydraulics in leaves as well as roots.

Species-specific coefficients for the response of *k*_total_ to flood duration (Eq. 3) were required for the SPA model to predict the contrasting patterns in *G*_s_ for flooded *M. grandiflora* and *Q. virginiana* (Fig. 5). Similarly, coefficients for the response of *k*_stem_ to water potential (i.e., vulnerability curves) vary greatly among sympatric tree species (McCulloh et al. 2019). Accounting for variation in hydraulic vulnerability underpins SPA modeling. Our results suggest that accounting for variation in the *k*_total_ response to flooding will enable SPA models to capture variation in flood performance among species and plant functional types. We used linear response curves for simplicity and because they fit well over our relatively short flood duration, but nonlinear responses, including recovery of *k*_total_ over time are likely to fit better for other species and flood scenarios.

We used a pragmatic SPA model that incorporates only the balance between water supply and demand to predict *G*_s_ (Sperry et al. 2016). Optimality-based *G*_s_ or *g*_s_ models that incorporate SPA dynamics along with parameters for carbon gain and storage perform better when light and CO_2_ levels vary (Wang et al. 2020; Potkay et al. 2025). Parameters for flood responses could be incorporated into such models. They would need to account for the effects of flooding on photosynthetic capacity along with *k*_total_ effects. The reduced Φ_PSII_ that we observed (Fig. 2) suggests that flooding caused photosynthetic decline and highlights the importance of incorporating the multiple impacts of flooding on plant physiological processes when projecting stomatal behavior under varying light and CO_2_ levels.

## Conclusions

Much of the progress made towards predicting tree stomatal responses has focused on water deficit conditions, however flood conditions also induce stomatal responses that are pervasive yet variable among tree species. Flooding and associated soil waterlogging are projected to increase in frequency and severity this century (Hirabayashi et al. 2013). The impacts of flooding on forests depend on the timing, duration, depth of floodwater, as well as forest composition and structure (Kozlowski 1997). Developing process-based models that project these impacts will help to predict forest dynamics and, if incorporated into land and Earth system models, global carbon and water cycling. Our results point towards accounting for flooding effects on *k*_total_ in SPA hydraulic modeling as a promising approach to project the stomatal responses that govern transpiration and influence plant growth and mortality during flood events.

## Supplementary Information

Below is the link to the electronic supplementary material.

Supplementary file1 (PDF 409 KB)

## Data Availability

The experimental data and analyses are publicly available on Zenodo at 10.5281/zenodo.17080340.
