# Supplementary material for: Hydraulic constraints to stomatal conductance in flooded trees
Source: Oecologia. 2025 Sep 10;207(10):154. doi: 10.1007/s00442-025-05789-y (PMC12423221; doi:10.1007/s00442-025-05789-y)
Supplement: Supplementary file 1 — Supplementary file1 (PDF 409 KB) [file 442_2025_5789_MOESM1_ESM.pdf]

Supporting Information
Hydraulic constraints to stomatal conductance in flooded trees
Brennan et al.

Methods S1

The Sperry model (Sperry et al. 2016) uses environmental conditions and plant traits to predict canopy vapor conductance, or, when boundary layer conductance is negligible, canopy-level stomatal conductance (G_s). We parameterized the model for the control plants with a single layer of sandy loam soil at soil water potential (Ψ_{soil}) = 0 MPa since pots were maintained near saturation. For roots, stems, and leaves, we fit vulnerability-curve parameters from measurements on stems (i.e., no vulnerability segmentation; Sperry et al. 2016). For *Magnolia grandiflora,* we used the vulnerability curve from Litvak et al. (2012). For *Quercus virginiana*, we found no vulnerability curve in the literature, so we measured stem vulnerability with the M-pneumatron method (Pereira et al. 2020). We collected three branches from trees growing on the Louisiana State University campus in Baton Rouge, LA, and air-dried them on a laboratory bench for 25 hours while periodically measuring leaf water potential (Ψ_{leaf}) on bagged leaves (putting them in equilibrium with stem water potential) and the volume of air discharged from the cut end of the branch with the M-pneumatron. We followed the protocol of Pereira et al. (2020) to construct vulnerability curves for each sample. We fit Weibull functions through the curves with the nls function in R and parameterized the model with the mean values among samples (Fig. S3, Table S1). Given the constraints of our vulnerability measurements, we performed a sensitivity analysis to test how varying vulnerability by 20% and including vulnerability segmentation (i.e., varying vulnerability among roots, stems, and leaves) would affect G_s prediction. We partitioned plant hydraulic resistance among roots, stem, and leaves based on measurements in the harvested control-treatment plants. For each plant, we calculated the proportion of hydraulic resistance in the roots by dividing total hydraulic conductance (k_{total}) by root hydraulic conductance (k_{root}; note that resistance is the inverse of conductance, so this is equivalent to dividing root hydraulic resistance by total hydraulic resistance). We then took the mean among samples to get the proportion of resistance in the root. One outlier sample from each species (value >2 SD from the mean) was removed for this calculation. We then divided the remaining proportion of plant hydraulic resistance evenly between stems and leaves. We used the VPD measurements taken with the LI600 porometer during g_s measurements for the air vapor pressure deficit (VPD) input. After all these parameters were set, we adjusted two unknown parameters to find the best fit between predicted and measured G_s, following the approach of Sperry et al. (2016) and Wolfe et al. (2016). The two unknown parameters were maximum k_{total} ($k_{total,max}$) and maximum *G.* Accurate values for these parameters are difficult to measure because $k_{total,max}$ and maximum G_s are reached only after continuous periods of high Ψ_{soil} and low VPD (Sperry et al. 2016). We adjusted values of $k_{total,max}$ and maximum *G* simultaneously to find the minimum mean absolute error between predicted and observed G_s in the timeseries of control-treatment plants. The hydraulic model assumes that light does not limit G_s (Sperry et al. 2016). This assumption is likely invalid in low-light conditions, so we excluded three cloudy days when midday photosynthetically active radiation (PAR) was >1SD below the mean (days 13, 27, and 41 in Fig. 1). All model parameter values are summarized in Table S1.

Once the parameters were fit to the control treatment plants, we tested the model on the flood treatment plants. We input the k_{root} reduction that we observed in the flood treatment by

reducing $k_{total,max}$ by the amount predicted from the linear models of k_{root} with flood duration (Table 1, Fig. 2k,l). We calculated the proportional difference in k_{root} between control and flooded plants at each timepoint as

$$\frac{k_{root,flood}}{k_{root,control}} = \frac{a + b_{flood} \times \text{duration}}{a + b_{control} \times \text{duration}} \quad \text{Eqn. S1}$$

where $k_{root,flood}$ and $k_{root,control}$ are k_{root} in the flood and control treatments, respectively; a, b_{flood}, and $b_{control}$ are the intercept and slope coefficients from the linear models on harvested plants (Table 1, Fig. 2k,l); and duration is the timespan of flooded days. The hydraulic model calculates maximum k_{root}, k_{stem}, and k_{leaf} ($k_{root,max}$, $k_{stem,max}$, $k_{leaf,max}$, respectively) from $k_{total,max}$ and the hydraulic resistance partitioning among roots, stems, and leaves (Sperry et al. 2016). We calculated $k_{total,max}$ in the flood treatment ($k_{total,max,flood}$) based on $k_{root,flood}/k_{root,control}$ as

$$k_{total,max,flood} = \frac{1}{\frac{1}{k_{root,max,control} \times \frac{k_{root,flood}}{k_{root,control}}} + \frac{1}{k_{stem,max,control}} + \frac{1}{k_{leaf,max,control}}} \quad \text{Eqn. S2}$$

where $k_{root,max,control}$, $k_{stem,max,control}$, and $k_{leaf,max,control}$ are the model outputs for maximum hydraulic conductance of the subscripted organ in the control treatment. This parameterization assumed that k_{total} reduction in flooded plants was caused solely by the effects of flooding on k_{root}.

In a separate test, we parameterized the model to account for the observed effects of flooding on k_{total}, not just the effects on k_{root}. Similar to the proportional reduction in k_{root} (Eqn. 1), we calculated the proportional reduction in k_{total} as

$$\frac{k_{total,flood}}{k_{total,control}} = \frac{a + b_{flood} \times \text{duration}}{a + b_{control} \times \text{duration}} \quad \text{Eqn. S3}$$

Where a, b_{flood}, and $b_{control}$ are the intercept and slope coefficients from the linear models on harvested plants (Table 1, Fig. 2g,h), and duration is the timespan of flooded days. We then calculated $k_{total,max,flood}$ as

$$k_{total,max,flood} = k_{total,max,control} \times \frac{k_{total,flood}}{k_{total,control}} \quad \text{Eqn. S4}$$

with $k_{root,flood}/k_{root,control}$ from eqn. 3. For *M. grandiflora*, the linear model predicted $k_{total,flood}$ <0 at flood duration >22 days (Table 1, Fig. 2g). Negative conductance is impossible; so for the two flood durations > 22 days (i.e., 24 and 26 days), we set $k_{total,flood} = 0$, which would predict $G_s = 0$. As with the control-treatment plants, for the flooded plants we set $\Psi_{soil} = 0$ MPa and input VPD with the timeseries of VPD measurements from the LI600 taken during g_s measurements.

We tested the model's performance in predicting G_s across flood and control treatments by fitting least squares linear regressions through observed versus model-predicted values. We used R^2 as an indicator of goodness of fit and the slope and intercept as indicators of bias.

Table S1 Inputs used for the hydraulic model of Sperry *et al.* (2016). Unless noted in the table, variables were set equally in the control and flood treatments. Unlisted variables were set at default values.

**Variable**	**Fitting method**	***M. grandiflora***	***Q. virginiana***
VPD (kPa)	Measured with a porometer (LI600, Campbell Scientific)	Varied at each timepoint	Varied at each timepoint
Soil water potential (MPa)	Single layer set at 0 MPa, representing soil at or near saturation	0 MPa	0 MPa
Rhizosphere hydraulic conductivity	Set for sandy loam soil	4510 kg hr^{-1} MPa^{-1}	4510 kg hr^{-1} MPa^{-}
Vulnerability curve parameters of root, stem, and leaf (MPa)	Measured on *Q. virginiana* stems (Fig. S3). For *M. grandiflora* stems, the Ψ_{50} value reported by Litvak et al. (2012) of -2.02 MPa was used to derive the vulnerability curve parameters. Root and leaf values were assumed to equal stem values (i.e., no vulnerability segmentation).	b = 2.3<br>c = 2.8<br><br>where<br>K/K_{max} = exp $(-((\Psi/b)^c))$	b = 4.112<br>c = 5.986<br><br>where<br>K/K_{max} = exp $(-((\Psi/b)^c))$
% resistance among roots, stems, and leaves at continuum k_{max}	Mean k_{total} / k_{root} from the harvested plants was used to calculate the % resistance in roots. It was assumed that the remaining % resistance was evenly split between stems and leaves.	93/3.5/3.5 for root/stem/leaf	81/9.5/9.5 for root/stem/leaf
Continuum k_{max}	Tuned for the control treatment by simultaneously adjusting k_{max} and maximum *G* to minimize the mean absolute error between observed and predicted G_s.	234 kg hr^{-1} MPa^{-1} m^{-2} for the control treatment. Flood treatment follows Eqns. S2 and S4.	141 kg hr^{-1} MPa^{-1} m^{-2} for the control treatment. Flood treatment follows Eqns. S2 and S4.
Maximum *G*	Tuned for the control treatment by simultaneously adjusting k_{max} and maximum *G* to minimize the mean absolute error between observed and predicted G_s.	12,000 kg hr^{-1} m^{-2} in both control and flood treatments	25,000 kg hr^{-1} m^{-2} in both control and flood treatments

Table S2 Logistic regression coefficients for the relationship between flood duration and presence/absence of hypertrophied lenticels. The coefficients fit within the following equation: proportion with hypertrophied lenticels = $\frac{1}{1+e^{-(a+b\times\text{duration})}}$, where duration is in days. Values shown are estimate ± SE. In parentheses are the *P* values from z tests for whether the estimate differs from zero.

Species	*a*	*b*
M. grandiflora	-4.17 ± 1.39 (0.003)	0.273 ± 0.092 (0.003)
Q. virginiana	-8.82 ± 4.09 (0.03)	0.375 ± 0.185 (0.04)

Table S3 Spearman correlations between physiological conditions in harvested plants. Values are listed for plants in both treatments combined and for the control and flood treatment in isolation. Symbols and units of measurement are as in Table 1. Significance levels for Spearman correlations are denoted as *italic, P < 0.05; **bold, P < 0.01; ***bold italic, P < 0.001.

	g_s	Φ_{PSII}	Ψ_{leaf}	k_{total}	k_{stem}	k_{root}	DM_{fine_root}
M. grandiflora							
Φ_{PSII}	0.82***<br>0.50*<br>0.75***	Combined<br>Control<br>Flood					
Ψ_{leaf}	-0.05<br>-0.38<br>0.06	0.01<br>-0.25<br>0.06	Combined<br>Control<br>Flood				
k_{total}	0.88***<br>0.16<br>0.85***	0.62***<br>-0.40<br>0.48**	0.13<br>0.16<br>0.27	Combined<br>Control<br>Flood			
k_{stem}	-0.06<br>-0.27<br>0.08	-0.07<br>-0.43<br>-0.10	0.24<br>-0.11<br>0.37*	0.00<br>0.06<br>0.14	Combined<br>Control<br>Flood		
k_{root}	0.42**<br>-0.27<br>0.52**	0.39**<br>-0.59*<br>0.52**	0.35*<br>0.58*<br>0.27	0.52***<br>0.41<br>0.60***	0.05<br>0.26<br>0.00	Combined<br>Control<br>Flood	
DM_{fine_root}	0.36*<br>0.05<br>0.22	0.21<br>-0.36<br>0.15	0.04<br>0.25<br>-0.04	0.32*<br>0.10<br>0.20	0.12<br>0.50*<br>-0.05	0.34*<br>0.23<br>0.36*	Combined<br>Control<br>Flood
Q. virginiana							
Φ_{PSII}	0.12<br>0.45<br>0.05	Combined<br>Control<br>Flood					
Ψ_{leaf}	0.35*<br>0.52*<br>0.25	0.31*<br>0.17<br>0.40*	Combined<br>Control<br>Flood				
k_{total}	0.56***<br>0.40<br>0.67***	-0.12<br>0.05<br>-0.28	0.44**<br>0.75***<br>0.18	Combined<br>Control<br>Flood			
k_{stem}	-0.19<br>-0.22<br>-0.19	-0.08<br>0.03<br>-0.06	0.15<br>0.20<br>0.22	0.06<br>0.18<br>-0.04	Combined<br>Control<br>Flood		
k_{root}	-0.06<br>0.00<br>-0.09	0.05<br>0.26<br>-0.10	-0.18<br>-0.23<br>-0.19	0.12<br>0.25<br>0.05	0.17<br>0.17<br>0.22	Combined<br>Control<br>Flood	
DM_{fine_root}	0.26<br>0.37<br>0.19	0.02<br>0.14<br>0.04	0.02<br>0.00<br>0.00	0.20<br>0.53*<br>0.11	0.01<br>-0.13<br>0.00	0.31*<br>0.56*<br>0.11	Combined<br>Control<br>Flood

Table S4 Path analysis model fits. For each species, four models were tested. The models varied in their inclusion of paths between flood duration and stomatal conductance (g_s) and between flood duration and total soil-to-leaf hydraulic conductance (k_{total}). Paths in gray font indicate that they were not included in the model. Columns are the number of parameters in the model (N. Parameters); the model test statistic (χ^2), its P value, and degrees of freedom (d.f.); the Akaike information criterion index corrected for the sample size (AICc); and the Akaike weight (AW). Models with bold AW were included in coefficient averaging.

Model	N. Parameters	χ^2 (P)	d.f.	AICc	AW
Magnolia grandiflora					
Flood duration → g_s<br>Flood duration → k_{total}	10	4.9 (0.43)	5	248.5	0.30
Flood duration → g_s<br>Flood duration → k_{total}	9	6.4 (0.38)	6	246.8	**0.70**
Flood duration → g_s<br>Flood duration → k_{total}	9	53.5 (0.00)	6	294.0	0.00
Flood duration → g_s<br>Flood duration → k_{total}	8	55.0 (0.00)	7	292.4	0.00
Quercus virginiana					
Flood duration → g_s<br>Flood duration → k_{total}	10	8.8 (0.12)	5	389.0	0.07
Flood duration → g_s<br>Flood duration → k_{total}	9	8.9 (0.18)	6	385.8	**0.31**
Flood duration → g_s<br>Flood duration → k_{total}	9	10.8 (0.09)	6	387.9	0.12
Flood duration → g_s<br>Flood duration → k_{total}	8	10.9 (0.14)	7	384.8	**0.51**

Table S5 Least squares linear regression coefficients of observed canopy-scale stomatal conductance (G_s) as a function of G_s predicted with a hydraulic model. The coefficients describe the regression lines shown in Fig. 5. Values shown are estimate ± SE. In parentheses are the *P* values from t tests of whether the estimate differs from zero.

Species	Flooding effect	Intercept	Slope	R^2
M. grandiflora	k_{root} reduction	-6520 ± 1853 (0.002)	1.69 ± 0.24 (5e-7)	0.69
M. grandiflora	k_{total} reduction	-187 ± 754 (0.81)	0.98 ± 0.11 (4e-9)	0.80
Q. virginiana	k_{root} reduction	-295 ± 4243 (0.95)	0.87 ± 0.33 (0.014)	0.24
Q. virginiana	k_{total} reduction	-262 ± 2435 (0.92)	0.98 ± 0.20 (9e-5)	0.51

Table S6 Results from the sensitivity analysis of predicted canopy-scale stomatal conductance (G_s) to hydraulic vulnerability and segmentation inputs. Hydraulic vulnerability was adjusted such that the water potential at 50% loss of hydraulic conductance (Ψ_{50}) was 20% lower than the values in Table S1; Ψ_{50} was 20% higher than the values in Table S1; and Ψ_{50} was 20% higher in the roots and leaves, but not the stem, representing hydraulic vulnerability segmentation. Intercept, slope, and R^2 are as described in Table S3; they describe the regression fit between predicted and observed G_s. ΔR^2 is the percent change in R^2 compared to the model fit with the vulnerability values listed in Table S1.

Species	Flooding effect	Intercept	Slope	R^2	ΔR^2 (%)
20% higher Ψ_{50}, no vulnerability segmentation					
M. grandiflora	k_{root} reduction	-6362 ± 1845 (0.002)	1.69 ± 0.24 (5e-7)	0.69	0
M. grandiflora	k_{total} reduction	-167 ± 754 (0.83)	0.99 ± 0.11 (4e-9)	0.80	0
Q. virginiana	k_{root} reduction	1561 ± 4513 (0.73)	0.71 ± 0.34 (0.05)	0.16	-33.3
Q. virginiana	k_{total} reduction	-29 ± 2622 (0.99)	0.95 ± 0.22 (3e-4)	0.46	-9.8
20% lower Ψ_{50}, no vulnerability segmentation					
M. grandiflora	k_{root} reduction	-5103 ± 2269 (0.04)	1.53 ± 0.29 (4e-5)	0.58	-15.9
M. grandiflora	k_{total} reduction	91 ± 963 (0.93)	0.93 ± 0.13 (5e-7)	0.69	-13.8
Q. virginiana	k_{root} reduction	-294 ± 4237 (0.95)	0.87 ± 0.33 (0.01)	0.24	0
Q. virginiana	k_{total} reduction	-253 ± 2433 (0.92)	0.98 ± 0.21 (9e-5)	0.51	0
20% higher Ψ_{50} in roots and leaves, vulnerability segmentation					
M. grandiflora	k_{root} reduction	-6313 ± 1933 (0.004)	1.67 ± 0.25 (1e-6)	0.66	-4.3
M. grandiflora	k_{total} reduction	-186 ± 754 (0.81)	0.99 ± 0.11 (4e-9	0.80	0
Q. virginiana	k_{root} reduction	-427 ± 4220 (0.92)	0.89 ± 0.33 (0.01)	0.25	+4.0
Q. virginiana	k_{total} reduction	-263 ± 2419 (0.91)	0.98 ± 0.20 (8e-5)	0.51	0

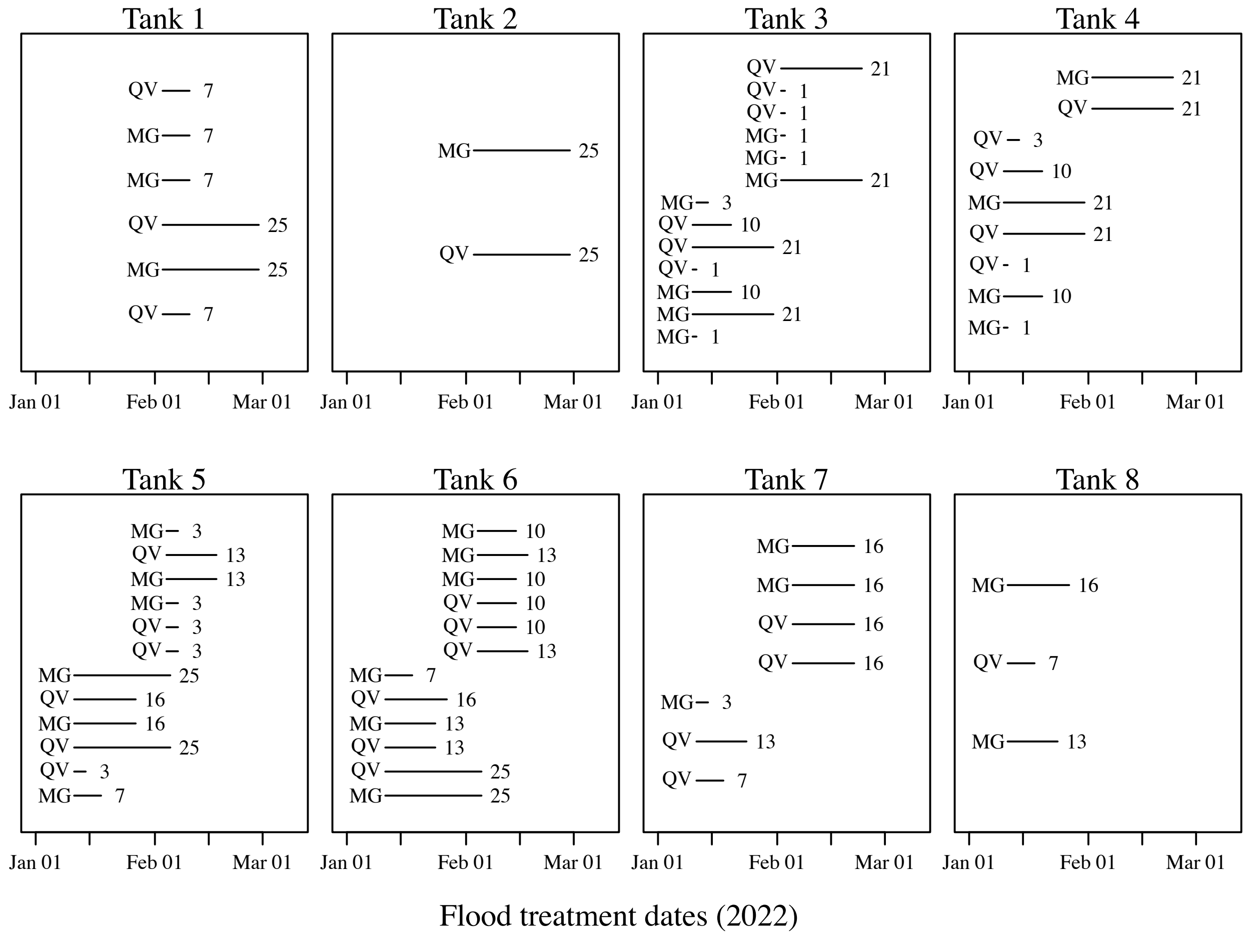

Figure S1 Schematic of experimental design for the flood treatment. Plants were distributed among 8 tanks (Tank 1–8). Within each tank, plants were exposed to flood conditions for various durations. Each line represents the dates (horizontal axis) that an individual plant was exposed to flood conditions. Lines labelled MG and QV represent *Magnolia grandiflora* and *Quercus virginiana*, respectively. Flood duration (days) is indicated to the right of each line.

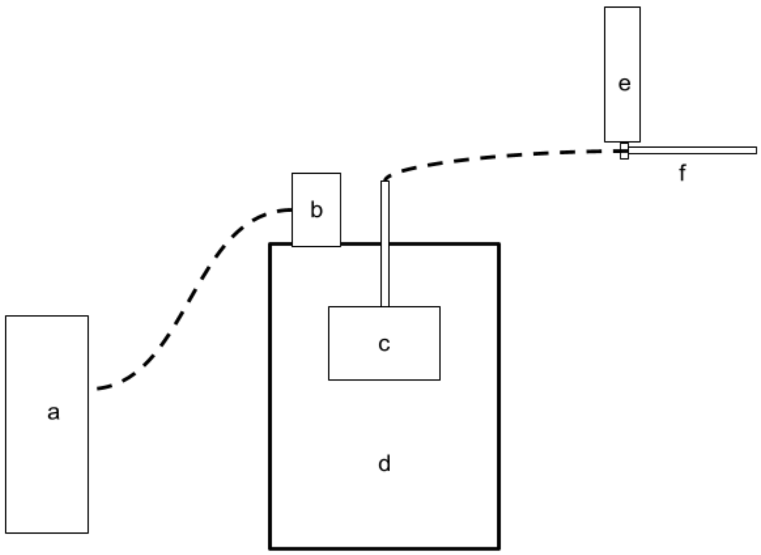

Figure S2 Diagram of the apparatus used to measure hydraulic conductance, modified from Kolb *et al.* (1996). (a) vacuum pump, (b) valve to regulate pressure and pressure gauge, (c) root or stem sample, (d) vacuum pressure chamber, (e) KCl solution reservoir, connected to graduated pipette (f) via three-way valve. Dashed lines represent tubing used to connect systems.

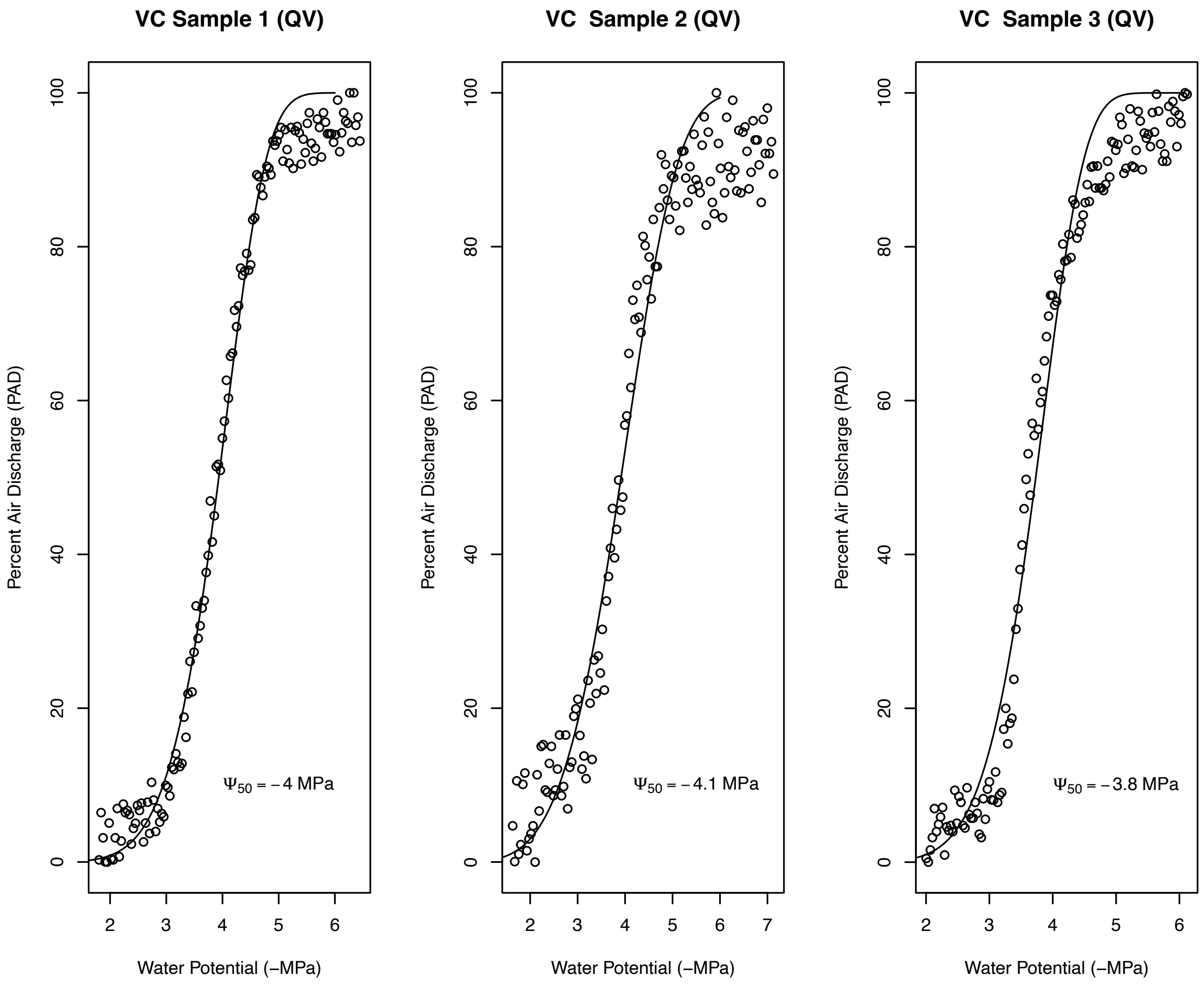

Figure S3 Vulnerability curves for three *Quercus virginiana* branches (Samples 1–3). Circles represent measured values of percentage of air discharge (PAD) and projected values of stem water potential. Curves represent Weibull functions fit through the points. The water potential at PAD = 50 (i.e., Ψ_{50}) is indicated.

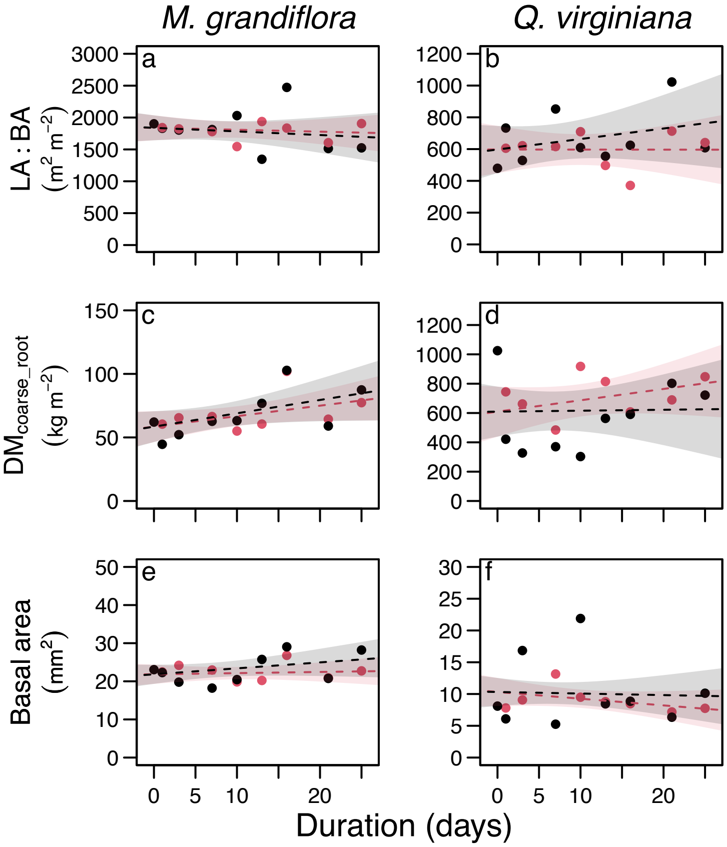

Figure S4 Leaf area to basal area ratio (LA:BA), coarse root dry mass per basal area (DM_{coarse_root}), and basal area among harvested plants in the flood and control treatments in *Magnolia grandiflora* (a, c, e) and *Quercus virginiana* (b, d, f). Red and black symbols represent the flood and control treatments, respectively. Each circle represents the mean of 2–4 plants. Lines represent outputs from linear models with the treatment by time interaction as the independent variable and the physiological condition as the dependent variable. Shaded regions are the 95% confidence intervals. Dashed lines indicate that the flood and control treatments did not have significantly different slopes ($P > 0.05$).

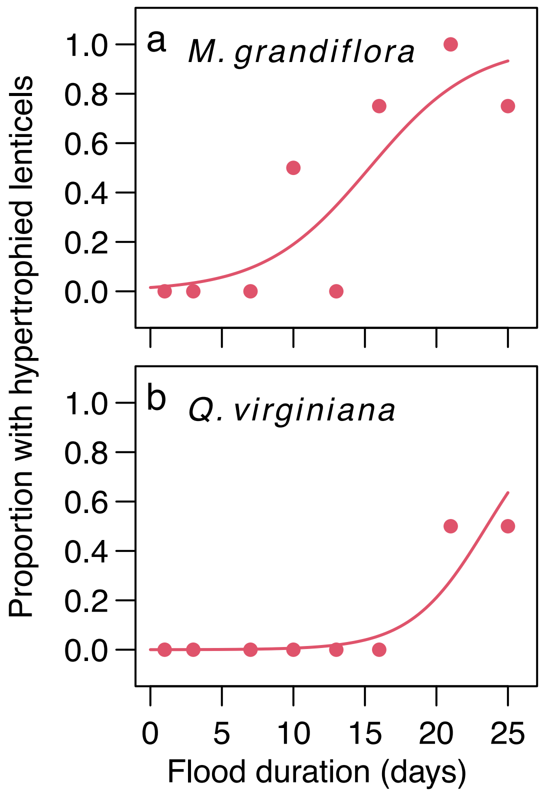

Figure S5 Relationship between flood duration and the proportion of plants that developed hypertrophied lenticels in (a) *Magnolia grandiflora* and (b) *Quercus virginiana.* Red lines were fit with logistic regression. Regression coefficients are summarized in Table S2.
